# Utility of the Modified 5-Items Frailty Index to Predict Complications and Mortality After Elective Cervical, Thoracic and Lumbar Posterior Spine Fusion Surgery: Multicentric Analysis From ACS-NSQIP Database

**DOI:** 10.1177/21925682221124101

**Published:** 2022-09-01

**Authors:** Gaston Camino-Willhuber, Jeffrey Choi, Fernando Holc, Sarah Oyadomari, Alfredo Guiroy, Hansen Bow, Sohaib Hashmi, Michael Oh, Nitin Bhatia, Yu-Po Lee

**Affiliations:** 1 Orthopaedic and Traumatology Department, Institute of Orthopedics “Carlos E. Ottolenghi,” 37533 Hospital Italiano de Buenos Aires, Buenos Aires, Argentina; 2 Department of Orthopaedics, 8788 University of California at Irvine, Orange, CA, USA; 3 School of Medicine, 8788 University of California Irvine, Irvine, CA, USA; 4 Elite Spine Health and Wellness Center, Fort Lauderdale, FL, USA; 5 Department of Neurosurgery, 8788 University of California Irvine, Orange, CA, USA

**Keywords:** modified frailty index, complications, spine surgery, readmission

## Abstract

**Study design:**

Retrospective review of multicentric data.

**Objectives:**

The modified 5-item frailty index is a relatively new tool to assess the post-operative complication risks. It has been recently shown a good predictive value after posterior lumbar fusion. We aimed to compare the predictive value of the modified 5-item frailty index in cervical, thoracic and lumbar surgery.

**Methods:**

The American College of Surgeons - National Surgical Quality Improvement Program (ACS-NSQIP) Database 2015-2020 was used to identify patients who underwent elective posterior cervical, thoracic, or lumbar fusion surgeries for degenerative conditions. The mFI-5 score was calculated based on the presence of 5 co-morbidities: congestive heart failure within 30 days prior to surgery, insulin-dependent or noninsulin-dependent diabetes mellitus, chronic obstructive pulmonary disease or pneumonia, partially dependent or totally dependent functional health status at time of surgery, and hypertension requiring medication. Multivariate analysis was used to assess the independent impact of increasing mFI-5 score on the postoperative morbidity while controlling for baseline clinical characteristics.

**Results:**

53 252 patients were included with the mean age of 64.2 ± 7.2. 7946 suffered medical complications (14.9%), 1565 had surgical complications (2.9%), and 3385 were readmitted (6.3%), 363 died (.68%) within 30 days postoperative (6.3%). The mFI-5 items score was significantly associated with higher rates of complications, readmission, and mortality in cervical, thoracic, and lumbar posterior fusion surgery.

**Conclusion:**

The modified 5-item frailty score is a reliable tool to predict complications, readmission, and mortality in patients planned for elective posterior spinal fusion surgery.

## Introduction

Posterior approaches to the cervical, thoracic, and lumbar spine allow direct neural decompression and provide stabilization with a lower risk of damaging vascular or visceral structures when compared with anterior approaches.^1-3^

Complications associated to fusion surgery are well documented, including surgical site infections, blood loss, and implant failure, which often lead to readmission and reoperation.^4-6^

Reoperation and readmission significantly increase hospital and healthcare cost.^7^ Patients’ comorbidities play an important role in postoperative complications, and different tools have been developed to assess the risk of complications and suitability for surgery.

Preoperative risk stratification processes are helpful to assess the risk of complications in orthopedic surgery with an intent to decrease patients’ burden and healthcare costs.^8^ In this regard, frailty indexes are a group of quantifiable tools designed to estimate complication rates based on patients’ comorbidities. The original frailty index encompassed a high number of items which made it difficult to use. Further modifications led to the development of the modified 11-items by Velanovich et al,^9^ which has been extensively used in spine surgery.^10-12^ Preoperative patients’ conditions recorded in the American College of Surgeons National Surgical Quality Improvement Program (ACS-NSQIP) Database had been used to gather these 11 items. However, changes in the database were thought to affect recording accuracy and therefore, proper preoperative assessment.^13^ Consequently, researchers designed a modified shorter version with 5 items to calculate the patients’ frailty.^14^ This modified 5-items frailty index has been recently employed as a parameter to predict the risk of complications in adult spinal deformity and lumbar fusion surgery showing consistent association with complications.^15,16^

However, the usefulness of the modified 5-items frailty index in other elective spine surgery, such as posterior cervical and thoracic fusion, has not been evaluated. Therefore, this study aims to analyze the impact and utility of this score system in predicting complications and readmission rates at 30 days postoperative period after posterior spinal fusion surgery.

## Methods

Posterior spinal fusion cases between January 2015 and December 2020 from the ACS-NSQIP database were gathered using the appropriate Current Procedure Terminology (CPT) codes: Posterior cervical (CPT-22590, 22 595, and 22 600), thoracic (CPT 22610), and lumbar fusion surgery (CPT-22612, 22 630, and 22 633). The ACS-NSQIP database consists of de-identified, validated information from multiple centers across the nation that report on surgical patients’ demographics, comorbidities, and 30-day postoperative measurable outcomes. The ACS NSQIP collects data on over 150 variables, including preoperative risk factors, intraoperative variables, and 30-day postoperative mortality and morbidity outcomes for patients undergoing major surgical procedures in both the inpatient and outpatient setting. The database automatically excludes patients below the age of 18 years. Institutional Review Board exemption was obtained from our institution and this study was deemed exempt from informed consent requirements.

Degenerative spinal conditions (spondylosis, degenerative spondylolisthesis, spinal stenosis) and deformities were included. Patients that required fusion surgery for a primary diagnosis of trauma, infection, or malignancy were initially excluded from the analysis. Patients with incomplete recording were also excluded.

The mFI-5 includes the following co-morbidities: 1) history of congestive heart failure, 2) presence of insulin-dependent or noninsulin-dependent diabetes mellitus, 3) history of chronic obstructive pulmonary disease or pneumonia, 4) partially dependent or totally dependent functional health status at time of surgery, and 5) presence of hypertension requiring medication (Table 1). Each item is assigned a ‘1’ if the comorbidity is present or ‘0’ when absent. The mFI-5 score is then calculated based on the sum of each of the 5 categories. Based on previous studies and for the purpose of our analysis, we classified the final mFI-5 scores of all patients into 3 groups: group 1 being patients with a score of 0, group 2 with a score of 1, and group 3 with a score of 2 or higher.

**Table 1.** Baseline Characteristics Based on Frail Status.

General	N = 53 252			
Variables	mFI-5 = 0	mFI-5 = 1	mFI-5 >= 2	*P* Value
Age (years)				**<.001**
Total	55.71 (SD 14.13)	65.11 (SD 11.04)	66.56 (SD 9.83)	
0-50	63 967 (34%)	2303 (10.1%)	704 (6.1%)	
51-65	7437 (39.5%)	8511 (37.2%)	4167 (36.1%)	
66-75	3635 (19.3%)	7986 (34.9%)	4498 (39%)	
>75	1371 (7.3%)	4082 (17.8%)	2162 (18.7%)	
Sex				**<.001**
Male	10 151 (53.9%)	11 780 (51.5%)	5516 (47.8%)	
Female	8688 (46.1%)	11 102 (48,5%)	6015 (52.2%)	
ASA grade				**<.001**
I	890 (4.7%)	44 (.2%)	17 (.1%)	
II	10 875 (57.7%)	8275 (36.2%)	1794 (15.6%)	
III	6675 (35.4%)	13 710(59.9%)	8614 (16.2%)	
IV	399 (2.1%)	847 (3.7%)	1098 (9.5%)	
V	0 (.0%)	6 (.0%)	8 (.1%)	
Any complication	2298 (12.2%)	3722 (16.3%)	2443 (21.2%)	**<.001**
Surgical Complication	434 (2.3%)	656 (2.9%)	475 (4.1%)	**<.001**
Superficial SSI	202 (1.1%)	273 (1.2%)	190 (1.6%)	**<.001**
Deep SSI	104 (.6%)	164 (.7%)	117 (1%)	**<.001**
Organ/space SSI	104 (.6%)	172 (.8%)	131 (1.1%)	**<.001**
Wound dehiscence	54 (.3%)	110 (.5%)	90 (35.4%)	**<.001**
Reoperation	629 (3.3%)	912 (4%)	557 (4.8%)	**<.001**
Medical complications				
Pneumonia	118 (.6%)	240 (37.6%)	281 (2.4%)	**<.001**
Unplanned intubation	49 (.3%)	134 (.6%)	141 (1.2%)	**<.001**
Pulmonary embolism	101 (.5%)	157 (.7%)	97 (.8%)	**.006**
Progressive renal insufficiency	9 (.0%)	45 (.2%)	50 (.4%)	**<.001**
Acute renal failure	5 (.0%)	21 (.1%)	40 (.3%)	**<.001**
Urinary tract infection	234 (1.2%)	426 (1.9%)	313 (2.7%)	**<.001**
CVA/stroke	21 (.1%)	50 (.2%)	58 (.5%)	**<.001**
Cardiac arrest	19 (.1%)	59 (.3%)	63 (.5%)	**<.001**
Myocardial infarction	34 (.2%)	105 (.5%)	112 (1.0%)	**<.001**
Bleeding transfusions	1433 (7.6%)	2308 (10.1%)	1399 (12.1%)	**<.001**
DVT/thrombophlebitis	130 (.7%)	208 (.9%)	103 (.9%)	**.034**
Sepsis	128 (.7%)	220 (1%)	186 (1.6%)	**<.001**
Septic Shock	26 (.1%)	60 (.3%)	81 (.7%)	**<.001**

All complications were analyzed between the 3 groups. Unadjusted comparisons using the Pearson c2 tests were used to assess for differences in complication rates between the 3 groups. Multivariate logistic regression was conducted to further analyzed each factor adjusted for age, sex, race, transfer status, admission status (inpatient vs outpatient), to separate the independent impact of increasing mFI-5 scores on postoperative outcomes. Results from regression analyses have been reported as adjusted odds ratio (OR) with their respective 95% confidence intervals and *P*-values. *P*-value of <.05 was considered significant. All statistical analysis was conducted using SPSS software, version 24 (IBM, Armonk, New York, USA).

## Results

53 252 patients met the inclusion criteria. 27 447 were male (51.6%) and 25 805 females (48.4%) with mean age of 64.2 ± 7.2. Baseline characteristics of our study population are illustrated in Table 1.

### Medical Complications

7966 patients suffered medical complications at 30 days postoperative period (14.9%), 1138 medical complications were observed in 9710 cervical fusion cases (11.7%), 1687 complications out of 3506 patients in the thoracic region (48.1%) and 5141 complications in 40 036 patients were found in the lumbar region (12.8%). The most common complications were bleeding requiring transfusions (n = 5150/64.5%), urinary tract infections (n = 973/12.2%), and pneumonia (n = 639/8%). Multivariate analysis showed a significant higher rate of medical complications in Group 3 in the cervical, thoracic and lumbar regions. A significant higher rate of medical complications was only observed in the lumbar region for Group 2. (Table 2)

**Table 2.** Multivariate Analysis of mFI-5 to Predict Medical Complications After Posterior Fusion.

Medical Complications						
Variable	Lumbar		Thoracic		Cervical	
	OR (95% CI)	*P* Value	OR (95% CI)	*P* Value	OR (95% CI)	*P* Value
mFI-5 = 1	1.25 (1.16-1.35)	**<.001**	.98 (.82-1.17)	.830	1.07 (.9-1.26)	.435
mFI-5> = 2	1.5 (1.38-1.64)	**<.001**	1.21 (.99-1.48)	.064	1.79 (1.51-2.14)	**<.001**
Pneumonia						
mFI-5 = 1	1.18 (.85-1.64)	.325	1.15 (.67-1.98)	.614	1.43 (.97-2.13)	.074
mFI-5> = 2	2.73 (1.97-3.79)	**<.001**	1.4 (.79-2.47)	.246	2.98 (2.02-4.4)	**<.001**
Unplanned intubation						
mFI-5=1	1.71 (1.03-2.84)	**.038**	1.01 (.42-2.44)	.975	1.67 (.97-2.85)	.064
mFI-5> = 2	2.94 (1.75-4.93)	**<.001**	2.38 (1.02-5.51)	**.044**	2.53 (1.46-4.36)	**.001**
Pulmonary embolism						
mFI-5 = 1	1.24 (.89-1.74)	.201	1.09 (.57-2.05)	.801	.82 (.46-1.47)	.503
mFI-5> = 2	1.7 (1.18-2.45)	**.005**	.9 (.42-1.94)	.793	.61 (.3-1.25)	.177
Progressive renal insufficiency						
mFI-5 = 1	3.58 (1.47-8.7)	**.005**	.83 (.13-5.28)	.848	3.69 (.44-30.61)	.227
mFI-5> = 2	6.01 (2.43-14.86)	**<.001**	4.46 (.9-22.24)	.068	6.85 (.83-56.33)	.073
Acute renal failure						
mFI-5 = 1	3.08 (.85-11.15)	.086	.61 (.18-2.1)	.437	1.05 (1.7-6.58)	.956
mFI-5> = 2	10.87 (3.12-37.82)	**<.001**	3.71 (1.07-12.89)	**.039**	4.05 (.8-20.57)	.091
Urinary tract infection						
mFI-5 = 1	1.32 (1.08-1.62)	**.007**	.85 (.52-1.39)	.516	.95 (.64-1.40)	.787
mFI-5> = 2	1.78 (1.42-2.22)	**<.001**	1.1 (.64-1.87)	.730	1.67 (1.13-2.48)	**.011**
CVA/stroke						
mFI-5 = 1	1.69 (.87-3.3)	.121	.33 (.43-2.6)	.295	1 (.38-2.63)	.993
mFI-5> = 2	3.09 (1.57- 6.11)	**.001**	1.17 (.2-6.75)	.860	2.73 (1.1-6.74)	**.030**
Cardiac arrest						
mFI-5 = 1	1.42 (.69-2.88)	.338	1.44 (.37-5.51)	.597	2.89 (.97-8.59)	.057
mFI-5> = 2	2.68 (1.30-5.51)	**.007**	3.13 (.85-11.48)	.085	4.52 (1.51-13.56)	**.007**
Myocardial infarction						
mFI-5 = 1	1.57 (.99-2.51)	.057	1 (.32-3.09)	.999	3.16 (1.08-9.23)	**.035**
mFI-5> = 2	2.53 (1.56-4.10)	**<.001**	2.8 (.97-8.09)	.057	8.09 (2.84-23.09)	**<.001**
Bleeding transfusions						
mFI-5 = 1	1.21 (1.11-1.32)	**<.001**	1.04 (.86 -1.25)	.681	1.13 (.89-1.44)	.311
mFI-5> = 2	1.34 (1.21-1.48)	**<.001**	1.17 (.95-1.45)	.145	1.78 (1.38-2.29)	**<.001**
DVT/thrombophlebitis						
mFI-5 = 1	1 (.75-1.33)	.992	.92 (.5-1.68)	.776	1.11 (.68-1.83)	.675
mFI-5> = 2	.8 (.56-1.15)	.227	.81 (.41 -1.63)	.563	1.09 (.62-1.92)	.770
Sepsis						
mFI-5 = 1	1.98 (1.44 -2.73)	**<.001**	.75 (.44-1.29)	.302	.94 (.6-1.47)	.793
mFI-5> = 2	3.16 (2.26-4.43)	**<.001**	.85 (.467 -1.54)	.589	1.46 (.91-2.34)	.112
Septic shock						
mFI-5 = 1	1.39 (.75-2.57)	.302	1.04 (.38-2.85)	.944	1.65 (.56-4.9)	.363
mFI-5> = 2	2.91 (1.57-5.39)	**.001**	2.07 (.77-5.58)	.151	5.34 (1.93-14.78)	**.001**

### Surgical Complications

1565 patients experienced surgical complications at 30-days postoperative period (2.9%). 326 complications were observed in the cervical spine (326/9710 = 3.3%), 161 in the thoracic (161/3506 = 4.6%) and 1078 in the lumbar spine (1078/40 036 = 2.7%). Superficial wound infection was the most (n = 665/42.5%), followed by organ space infections (n = 407/26%), and deep infections (n = 385/24.6%).

On multivariate analysis, mFI-5 > = 2 was a significant predictor for any surgical complication in the cervical, thoracic, and lumbar region. mFI-5=1 was a significant predictor for lumbar surgery. Regarding the types of surgical complication, superficial, deep and organ space infections significantly correlated with mFI-5> = 2 in the cervical and lumbar spine but not in the thoracic spine.

Regarding wound dehiscence, mFI-5> = 2 was predictor in all spinal regions (see table 3)

**Table 3.** Multivariate Analysis of Surgical Complications According to Frailty.

Surgical Complications						
Variable	Lumbar		Thoracic		Cervical	
	OR (95% CI)	*P* Value	OR (95% CI)	*P* Value	OR (95% CI)	*P* Value
mFI-5 = 1	1.28 (1.11-1.48)	**.001**	1.2 (.8-1.8)	.385	1.16 (.88-1.53)	.303
mFI-5> = 2	1.72 (1.46-2.02)	**<.001**	1.98 (1.31-2.98)	**.001**	1.92 (1.44-2.55)	**<.001**
Superficial SSI						
mFI-5 = 1	1.31 (1.04-1.65)	**.022**	.79 (.37-1.68)	.543	1.36 (.88-2.08)	.164
mFI-5> = 2	1.89 (1.46-2.44)	**<.001**	1.36 (.63-2.93)	.427	1.67 (1.03-2.71)	**.039**
Deep SSI						
mFI-5 = 1	1.42 (1.05-1.92)	**.021**	1.05 (.44-2.49)	.919	1.04 (.52-2.07)	.907
mFI-5> = 2	1.5 (1.05-2.13)	**.024**	1.56 (.63-3.85)	.337	3.175 (1.68-6.01)	**<.001**
Organ/space SSI						
mFI-5 = 1	1.51 (1.1 -2.08)	**.011**	1.2 (.6-2.39)	.603	1.57 (.88-2.82)	.130
mFI-5> = 2	2.05 (1.44-2.91)	**<.001**	1.68 (.81-3.5)	.162	2.66 (1.44 -4.92)	**.002**
Wound dehiscence						
mFI-5 = 1	1.71 (1.1-2.64)	**.016**	4.81 (1.04-22.25)	**.045**	1.22 (.66 -2.28)	.524
mFI-5> = 2	2.08 (1.27-3.34)	**.003**	9.19 (1.97-42.78)	**.005**	2.38 (1.28-4.43)	**.006**

### Readmission and Mortality

3385 patients were readmitted within 30-days postoperatively (6.3%). Cervical readmission was observed in 25.7% (n = 872), thoracic region 43.3% (n = 1466), and lumbar 30.9% (n = 1047). Multivariate analysis showed that mFI-5> = 2 was a significant predictor for unplanned readmission in the cervical, thoracic and lumbar regions. mFI-5 = 1 was also a significant predictor for lumbar readmission (Table 4).

**Table 4.** Multivariate Analysis of Unplanned Readmission and Mortality.

Variable	Lumbar		Thoracic		Cervical	
	OR (95% CI)	*P*	OR (95% CI)	*P*	OR (95% CI)	*P*
30-day readmission						
mFI-5 = 1	1.31 (1.17-1.46)	**<.001**	1.09 (.82-1.45)	.561	1.19 (.98-1.45)	.084
mFI-5> = 2	1.78 (1.58-2.01)	**<.001**	1.73 (1.28-2.35)	**<.001**	1.65 (1.33-2.03)	**<.001**
30-day mortality						
mFI-5 = 1	1.52 (.9-2.58)	.115	1.03 (.49-2.16)	.932	1.35 (.84-2.18)	.218
mFI-5> = 2	2.77 (1.63-4.71)	**<.001**	2.37 (1.17-4.79)	**.016**	1.72 (1.04-2.82)	**.033**

363 patients died at 30 days postoperative period (.68%). Multivariate analysis showed a significant higher mortality risk in Group 3 when compared with Group 1. There was no significant difference in mortality between Group 1 and Group 2 (Table 4).

## Discussion

The modified 5-items frailty index has shown to predict complications after posterior lumbar fusion surgery. To our knowledge, this is the first study that analyzed the utility of mFI-5 to predict complications after elective posterior cervical, thoracic, and lumbar fusion surgery. The modified 5 items frailty index was shown to be a significant predictor of medical and surgical complications after elective posterior spinal fusion surgery. mFI-5 score of 2 or higher was significantly associated with higher rates of unplanned readmission and mortality in all spinal regions. Moreover, mFI-5 score of 1 showed an association with a higher rate of unplanned readmission and surgical complications in the lumbar region but not in the thoracic or cervical region.

The m-FI-5 score has been previously shown to be a predictor of post-operative complications in general surgery^17,18^ and orthopaedic surgery.^19-21^ Chan et al recently reported the usefulness of the modified frailty score to predict complications after lumbar degenerative spondylolisthesis treated with posterior approach in 15 658 patients.^22^ Conversely, Elsamadicy et al showed no association between frailty status assessed by the mFI-5 score and the risk of complications and readmission in lumbar spondylolisthesis 5296 patients treated with posterior decompression and fusion.^23^ Our study supports the results from Chan et al, and this could be explained by the higher number of patients analyzed in our study.

Yagi et al^24^ compared the 11-item and 5-item frailty scores in 281 adult spinal deformity patients and observed similar complication predictions between 2 scores.

Regarding cervical spine fusion, Shin et al evaluated the role of mFI-11 items to predict major complications after ACDF and Posterior cervical fusion and showed significant associations.^25^ In summary, both mFI-5 and mFI-11 scores appear to serve similar function with the advantage of the mFI-5 score being easier and faster, therefore more practical to calculate.

To our knowledge, this is the first study to analyze the role of mFI-5 in posterior cervical and thoracic fusion surgery. Our data suggest similar tendency to predict medical and surgical complications in cervical and lumbar posterior surgery and medical complications in thoracic surgery. mFI-5 score was not shown to be a significant predictor of superficial, deep and organ space complications in posterior thoracic fusion, but it was significant for wound dehiscence. This could be due to lower number of cases in the thoracic region compared with cervical and lumbar surgeries or could be related to other factors not properly identified in the study. Medical complications, unplanned readmission, and mortality rates were significantly associated with a higher score in thoracic region, showing similar impact of frailty in all 3 regions.

Our study has some limitations. First, this is a retrospective study with inherent limitations. The recording of adverse events is limited to 30 days postoperative as standardize by the NSQIP guidelines. Therefore, long-term complications are not being captured. Another limitation of this study is that we did not stratified diagnoses but analysis was solely based on anatomic region. Also, we did not take the number of levels performed in the consideration.

This is the largest study that analyzed the modified 5 items frailty index, and the first to compare cervical with thoracic and lumbar fusion. From the epidemiologic standpoint, our results can be used as a frame to demonstrate the need of preoperative optimization and complication risk and mortality assessment based on preoperative frailty state.

## Conclusion

The modified 5 items frailty index has been shown to be a strong predictor of 30-day medical complications and mortality after elective posterior fusion in the cervical, thoracic, and lumbar spine and a strong predictor for surgical complications in the cervical and lumbar spine. This preoperative scoring system could represent an additional and reliable tool to assess the risk of short-term complications after elective posterior spinal fusion surgery.
